# Nuclear Receptor PPARα as a Therapeutic Target in Diseases Associated with Lipid Metabolism Disorders

**DOI:** 10.3390/nu15224772

**Published:** 2023-11-13

**Authors:** Ping Hu, Kaiqi Li, Xiaoxu Peng, Yufei Kan, Hao Li, Yanli Zhu, Ziyu Wang, Zhaojian Li, Hao-Yu Liu, Demin Cai

**Affiliations:** 1 College of Animal Science and Technology, Yangzhou University, Yangzhou 225009, China; pinghu@yzu.edu.cn (P.H.); mx120220853@stu.yzu.edu.cn (K.L.); 211902226@stu.yzu.edu.cn (X.P.); kanyufei2023@163.com (Y.K.); 17633532469@163.com (H.L.); 17612808232@163.com (Y.Z.); 211902331@stu.yzu.edu.cn (Z.W.); 007919@yzu.edu.cn (Z.L.); 2 International Joint Research Laboratory in Universities of Jiangsu Province of China for Domestic Animal Germplasm Resources and Genetic Improvement, Yangzhou 225009, China

**Keywords:** PPARα, lipid metabolism, metabolic disorders, diabetes-related diseases, cell senescence, cancer

## Abstract

Lipid metabolic diseases have substantial morbidity and mortality rates, posing a significant threat to human health. PPARα, a member of the peroxisome proliferator-activated receptors (PPARs), plays a crucial role in lipid metabolism and immune regulation. Recent studies have increasingly recognized the pivotal involvement of PPARα in diverse pathological conditions. This comprehensive review aims to elucidate the multifaceted role of PPARα in metabolic diseases including liver diseases, diabetes-related diseases, age-related diseases, and cancers, shedding light on the underlying molecular mechanisms and some regulatory effects of natural/synthetic ligands of PPARα. By summarizing the latest research findings on PPARα, we aim to provide a foundation for the possible therapeutic exploitation of PPARα in lipid metabolic diseases.

## 1. Introduction

Metabolic diseases encompass a diverse array of medical conditions resulting from dysregulation in the body’s metabolic pathways. These disorders disrupt the delicate balance of nutrient utilization and energy production, giving rise to a range of health issues. Metabolic diseases include various types, such as liver diseases, diabetes-related diseases, age-related diseases, and others, each characterized by unique manifestations and underlying metabolic disturbances [1,2]. Notably, extensive research has increasingly recognized dysregulated metabolism as a hallmark of cancer [3,4]. Cancer cells exhibit significant alterations in cellular energy utilization and nutrient uptake, which are central to tumor growth and survival [5]. In light of this, cancer is now regarded as one of the metabolic diseases, intertwining its pathogenesis with metabolic imbalances [6]. Recognizing cancer as a metabolic disease opens new avenues for research and therapeutic interventions. Integrating metabolic considerations into cancer treatment strategies holds tremendous potential for improving patient outcomes and enhancing the overall efficacy of cancer therapies.

Peroxisome proliferator-activated receptors (PPARs) constitute a class of nuclear receptors that play a pivotal role in regulating physiological homeostasis, including lipid and carbohydrate metabolism in various tissues [7]. There are three distinct subtypes of PPAR: PPARα, PPARβ/δ, and PPARγ [8]. These three isotopes differ from each other in terms of their tissue distributions, ligand specificities, and physiological roles. The PPARα subtype is abundant in highly active metabolic tissues such as the liver, heart, muscle, kidney, brown adipose tissue, and vascular wall cells, including endothelial cells, smooth muscle cells, and macrophages [8]. PPARα is crucially involved in lipid metabolic homeostasis [9,10]. PPARα activation triggers the change in expression of multiple genes, encompassing the lipoprotein lipase gene, which facilitates the liberation of fatty acids from lipoprotein particles, and genes responsible for encoding the fatty acid translocase CD36 and fatty acid-binding proteins [11]. Early studies on CD36 primarily focused on its involvement in lipid metabolism and atherosclerosis. However, recent research has shown that CD36 plays a promoting role in the metastasis of oral cancer and breast cancer [12]. It is believed that CD36 helps tumor cells to uptake fatty acids from the surrounding environment, thus gaining the energy required for metastasis. Given that PPARα regulates the expression of CD36 in fatty acid metabolism, it may be possible in the future to utilize PPARα to inhibit CD36 expression or block its function, thereby providing a potential therapeutic approach for inhibiting cancer cell metastasis. Moreover, PPARα plays a role in governing genes linked to mitochondrial fatty acid β-oxidation, including those related to carnitine palmitoyl transferase one and medium chain-acyl-CoA dehydrogenase, contributing to the coordination of fatty acid β-oxidation [13,14]. Furthermore, PPARα has been shown to inhibit cell proliferation, induce cell cycle termination, and induce apoptosis in multiple cancer cells, which promotes intercellular adhesion and mitigates the inflamed state of the tumor microenvironment [15,16]. As a nuclear receptor, PPARα forms dimers by binding to specific ligands, and further regulate transcription by binding to the target DNA sequences. This is the primary mechanism through which PPARα participates in various regulatory processes. It is also a major reason why it can serve as a therapeutic target.

In this review, we aim to provide a comprehensive overview of the roles of PPARα in lipid metabolic diseases, such as liver diseases, diabetes-related diseases, growth disorders, and even cancers, in the context of metabolic diseases. By integrating the latest research findings, we hope to shed light on the potential therapeutic implications of targeting PPARα in the prevention and treatment of metabolic disorders. Understanding the complex interactions between PPARα and metabolic diseases holds promise for the development of innovative therapeutic strategies and personalized medicine approaches for these devastating diseases.

## 2. PPARα Is a Critical Player in Nonalcoholic and Alcoholic Liver Diseases

Liver diseases are often intricately linked to metabolic imbalances and are a major global health concern. The liver serves as a metabolic hub, regulating carbohydrate, lipid, and protein metabolism, and its dysfunction can result in devastating consequences for overall health. Nonalcoholic fatty liver disease (NAFLD) is a prevalent global public health concern and a chronic liver metabolic disorder affecting approximately 30% of adults worldwide [17]. It is characterized by the accumulation of neutral lipids forming lipid droplets in liver cells [18]. Moreover, alcohol-related liver disease (ALD) includes a range of disorders of different severity and is one of the most prevalent types of liver disease worldwide [19]. Current research demonstrates that activated PPARα is currently undergoing clinical trials in liver disease [20,21,22,23]. Therefore, this section provides a comprehensive review of the role of PPARα in NAFLD and ALD.

### 2.1. Nonalcoholic Fatty Liver Disease

NAFLD can progress from simple steatosis to non-alcoholic steatohepatitis (NASH), ultimately leading to cirrhosis and hepatocellular carcinoma. NAFLD has reached epidemic levels in some areas of East Asia [24,25]. It should be noted that the deletion of PPARα, specifically in the liver, contributes to NAFLD in obesity [26]. The researchers performed a knockout of PPARα in the whole body (Pparα^−/−^) and specifically in the liver of mice (Pparα^hep−/−^). They observed elevated levels of total cholesterol, low-density lipoprotein (LDL) cholesterol, and high-density lipoprotein (HDL) cholesterol in the blood of Pparα^hep−/−^ mice compared to Pparα^−/−^ and WT mice after high-fat diet (HFD) feeding [27]. Additionally, both Pparα^hep−/−^ and Pparα^−/−^ mice exhibited increased levels of aspartate transaminase (AST) and alanine transaminase (ALT), which imply liver injury in high-fat diet (HFD)-induced obesity [27]. In addition, previous observations confirm the role of hepatocyte PPARα in repressing the expression of inflammatory genes, such as those involved in the NF-kappa B pathway [28]. Specifically, under methionine-deficient and choline-deficient (MCD) diet conditions, GPS2-knockout mice showed reduced activities of AST and ALT, decreased hepatic steatosis, and improved hepatic fibrosis, thereby preventing the development of NAFLD and other associated disorders [29]. In addition, cyclic adenosine monophosphate (AMP)-responsive element-binding protein H (CREBH) is an endoplasmic reticulum-anchored transcription factor that exhibits selective expression in the liver and small intestine, which plays a crucial role in the development of NAFLD [30]. Zhang et al. revealed that CREBH expression was either reduced or remained unglycosylated in NAFLD, resulting in decreased expression of PPARα [31]. The process of *N*-glycosylation of CREBH by glycosyltransferase V (GnT-V) and other factors impairs the recognition of the cyclic adenosine monophosphate-responsive element (CRE) in the promoter region. This, in turn, facilitates the interactions between CREBH/PPARα and CREBH/SCD-1, subsequently upregulating the expression of CREBH and PPARα. Consequently, this mechanism contributes to the improvement or prevention of NAFLD [31].

**Figure 1:**
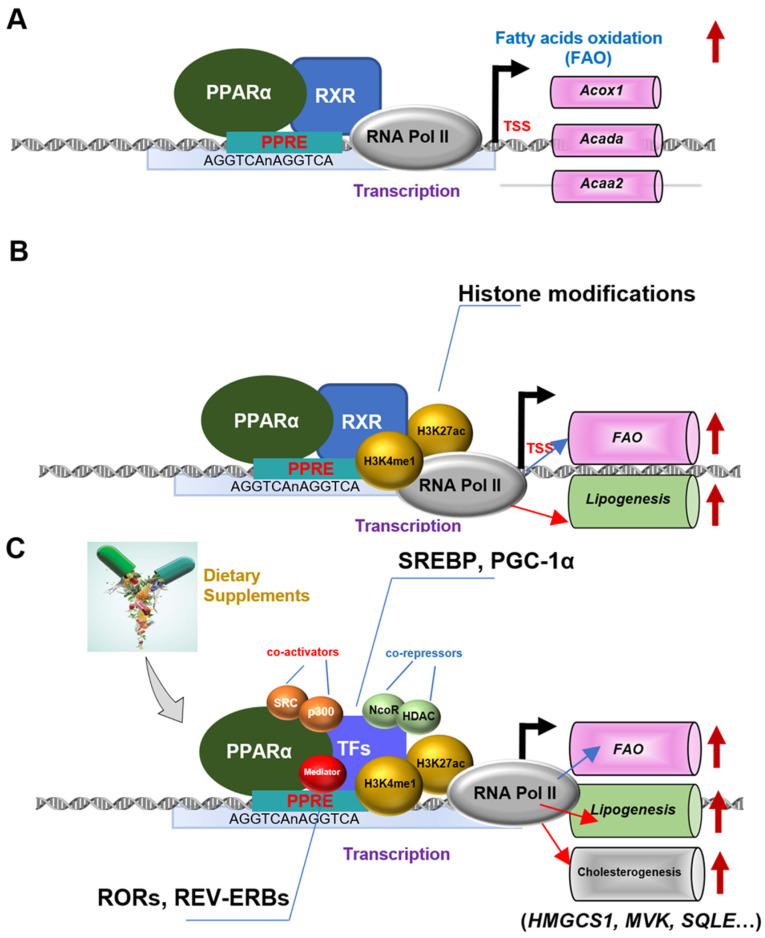
Schematic illustration depicting the molecular mechanisms of PPARα modulate hepatic steatosis. (**A**): PPARα binds to specific PPREs as a heterodimer with RXR and then regulates fatty acids oxidation under physiological condition. (**B**): PPARα-mediated the actions of dietary interferences on lipid biosynthesis and fatty acid oxidation by epigenetic modulations. (**C**): The regulation mechanism of PPARα in directing abnormal lipogenesis, fatty acids oxidation, and cholesterogenesis via the recruitment of histone marks and co-factors when compose a novel and “atypical” transcription factors crosstalk at both the gene promoter and the enhancer.

Interestingly, a more recent study reported contrasting results, as they found that mice with intestine-specific PPARα deletion (PPARα^∆IE^) fed with HFD showed reduced serum ALT, TC, and NEFA levels, as well as significant reductions in liver inflammation and fibrosis [26]. The study also discovered that a high-fat diet (HFD) can activate intestinal PPARα in mice, which, in turn, induces the expression of fatty acid binding protein 1 (FABP1) and facilitates the uptake of fatty acids by intestinal PPARα. Moreover, activation of PPARα ameliorates liver fibrosis and inhibits the function of hepatocyte-specific G-protein pathway suppressor 2 (GPS2), alleviating the development of diet-induced steatosis and fibrosis and causing activation of lipid catabolic genes [32,33]. In agreement with this notion, our recent studies highlight a context-specific modulation that PPARα signaling activation enhances lipid deposition in the liver [32,33]. It is well known that PPARα activation predominantly facilitates fatty acids oxidation through upregulating the genes Acads and Acaa2, as shown in Figure 1A, under physiological conditions. However, “atypical” PPARα actions might be stimulated under the pathophysiological status, in which lipid biosynthesis pathway is firstly activated by PPARα and the genes Acaca, Fasn, and Scd are up-regulated. The upregulation of the expression of these three genes implies a regulatory effect different from that of PPARα mentioned in the previous context under normal physiological conditions, which means it will direct abnormal lipogenesis, fatty acids oxidation and cholesterogenesis. Given the role of “McGarry’s Vicious Cycle”, a cycle in which insulin resistance leads to a self-reinforcing negative regulatory loop, where elevated insulin levels exacerbate diseases like steatosis, it will further decrease sensitivity to insulin and increase serum insulin levels to sustain the negative loop [34]. This kind of loss of white adipose tissue and the increment of blood insulin and glucose contribute to lipid deposition in the liver called a “lipid stealing”. Additionally, the expression of genes Acat2, Hmgcs, Hmgcr, Mvd, Sqle, and Dhcr 7/24 involved in cholesterol *de novo* synthesis are also increased when PPARα is abnormally hyper-expressed. Intriguingly, some organics like chlorogenic acid can alleviate hypercholesterolemia induced by a high-cholesterol diet by upregulating the expression of PPARα gene [35]. The above research reveals the complex relationship between PPARα and cholesterol synthesis and metabolism. Mechanistically, because of the betray of PPARα in response to dietary intervention, PPARα cooperates with classic lipid metabolic drivers like SREBPs to reprogram the genes of their specific binding. In this process, some mediators, including nuclear receptors RORs and REV-ERBs, are recruited and then generate a newly reconstituted transcriptional complex (Figure 1B). Importantly, co-factors and histone marks are enrolled in the regulation. To our knowledge, co-activators SRCs and p300 together with co-repressors NcoRs and HDACs are critical for PPARα-mediated lipid metabolic disorders (Figure 1C). Of note, H3k4me1 and H3K27ac represent pivotal epigenetic modulation to accelerate a conformational change in the PPARα, performing transcriptional recognition in the lipid metabolic genes specific to their enhancer regions. It is worth mentioning that PPARα-controlled lipid abnormal metabolism, in context and cell-type-specific patterns, may possibly be utilized for the treatment of lipid metabolic illnesses caused by dietary interferences. The therapeutic strategy of using nutrients such as fatty acids and their metabolic products to regulate PPARα has solid theoretical support. Recent studies have shown that a diet high in castor oil, which is rich in erucic acid (a long-chain fatty acid), activates PPARα and enhances peroxisome β-oxidation capacity, suggesting that erucic acid may serve as a potential ligand for PPARα. Treating Fao cells from rats with fungal lipid extracts rich in branched-chain fatty acids (Conidiobolus heterosporous) increased the mRNA levels of PPARα target genes Acox1, Cyp4a1, Cpt1A, and Slc22A5, strongly indicating that branched-chain fatty acids also serve as potent PPARα agonists. Taken together, these findings confirm that peroxisome β-oxidation substrates are potent PPARα ligands capable of regulating the expression of a range of lipid-metabolizing enzymes to maintain lipid homeostasis and alleviate the toxic effects of excessive long-chain fatty acids and branched-chain fatty acids [36].

### 2.2. Alcohol-Related Liver Disease

Alcohol abuse remains a leading cause of liver disease and liver disease-related mortality [37,38]. Prolonged and excessive alcohol consumption can lead to liver fat accumulation, inflammation, and other detrimental effects, collectively known as ALD [39]. Among the three alcohol-metabolizing enzymes, the catalase pathway shows potential for reducing reactive oxygen species (ROS) and achieving a relatively less harmful alcohol-elimination process by breaking down H_2_O_2_ into water and oxygen [40]. As a key regulator of peroxisomal biogenesis and homeostasis, PPARα directly influences the expression of peroxisomal catalase [33,41]. Additionally, PPARα plays a crucial role in the regulation of the NAD^+^ biosynthesis pathway, which is closely related to the function, biosynthesis, and metabolism of mitochondria and is also involved in alcohol metabolism [21]. In terms of mitochondrial metabolism, the expression of Peroxisome Proliferator-Activated Receptor Gamma Coactivator 1-alpha(PGC-1α), which is involved in regulating the quantity and function of mitochondria, enhancing cellular oxidative capacity and resilience, is regulated by PPAR-α and helps in increasing the activity and efficacy of PPAR-α. PPAR-α and PGC-1α closely collaborate by regulating common target genes, participating in lipid metabolism, and preventing oxidative stress and other physiological processes. The interaction between them is crucial for maintaining cellular energy balance, regulating lipid metabolism, and protecting cells from oxidative stress damage. An existing study has shown that in mice, the deficiency of Atgl(patatin-like phospholipase domain containing 2) induces a decrease in the mRNA levels of PPAR-α, leading to reduced expression of PGC-1α and severe impairment of mitochondrial substrate oxidation and respiration [42]. Atgl is one of the synthetic enzymes involved in the production of hydroxy-fatty acids, so its regulation of PPARα mRNA levels may be associated with the interaction between fatty acids and PPARα. Yue et al. demonstrated that the protein level of PPARα in the liver of patients with severe alcoholic hepatitis was decreased, accompanied by increased serum levels of ALT and AST, hepatocyte necrosis, and degeneration [21]. Remarkably, mice treated with a PPARα agonist exhibited a remarkable 69% decrease in serum ethanol concentrations and a striking reduction of over 95% in hepatic ethanol levels, along with lowered acetaldehyde levels and efficient mitigation of alcohol-induced H_2_O_2_ accumulation in both serum and liver, ultimately restoring these levels to a state of normalcy [21].

## 3. Therapeutic Approaches to Diabetes-Related Diseases via PPARα-Dependent Pathways

Diabetes mellitus, classified as a metabolic disorder, is predominantly marked by aberrations in carbohydrate, lipoprotein, and lipid metabolism. These abnormalities culminate in chronic hyperglycemia, often accompanied by complications stemming from either insulin deficiency or insulin resistance within the body [43]. Diabetes-associated maladies historically encompassed macrovascular disorders, exemplified by heart disease, stroke, and peripheral arterial disease. Concurrently, microvascular afflictions comprise diabetic retinopathy, peripheral neuropathy, and kidney disease [44]. PPARα manifests its presence in organs impacted by diabetic disease, with its expression intricately governed within these specific tissues. This phenomenon hints at the novelty of PPARα as a potential target for diabetic maladies [45,46].

### 3.1. Heart Disease

Diabetes mellitus-induced heart disease, notably encompassing diabetic cardiomyopathy, poses a significant medical challenge characterized by treatment complexity. Diabetes mellitus elevates the susceptibility to heart failure and concurrently diminishes cardiac myocyte function. These effects are intricately interwoven with alterations in cardiac mitochondrial energy metabolism [47]. Central attributes of diabetic cardiomyopathy encompass atypical cardiac myocyte contraction and perturbed fuel flux, marked by attenuated glucose oxidation and markedly augmented fatty acid oxidation. This shift is interrelated with diminished energetic efficiency owing to impaired mitochondria [48]. PPARα regulates cardiac energy and lipid metabolism, notably playing a pivotal role in mitochondrial FA β-oxidation, which is essential for fuel generation in the heart. This role is achieved through the transcriptional activation of carnitine palmitoyl transferase 1 [49]. The heart primarily relies on mitochondrial fatty acid oxidation (FAO) for ATP generation yet possesses metabolic flexibility to transition towards alternative energy substrates, particularly glucose. This transition in substrate preference is observed during myocardial ischemia, cardiac hypertrophy, and heart failure [50]. However, glucose possesses the capacity to downregulate the expression of PPARα, consequently resulting in diminished FAO levels [51]. In addition to their direct anti-inflammatory and anti-atherosclerotic effects on arterial walls, PPARα and its agonists have a favorable impact on lipid and lipoprotein metabolism [7]. PPARα potentially modifies lipid metabolism through various mechanisms, facilitating the transfer of fatty acids (FAs) into mitochondria. Furthermore, PPARα binds to both synthetic and natural ligands, resulting in a reduction in the PPARα receptor’s half-life. This process ultimately fine-tunes lipid metabolism to counteract dyslipidemia, a significant risk factor for cardiovascular diseases.

### 3.2. Diabetic Retinopathy

Diabetic retinopathy (DR) is a leading cause of blindness among working-age adults, with a global incidence of up to 35% in individuals with diabetes [52]. The prevalence of DR is steadily increasing, posing a significant threat to human health [53]. Disturbances in blood components in diabetic patients contribute to endothelial cell dysfunction, leading to the disruption of the blood–retinal barrier [54]. Increased retinal vascular permeability, resulting from blood–retinal barrier damage, is a key pathological feature in the early stages of DR [55]. Vascular endothelial growth factor (VEGF) plays a crucial role in promoting angiogenesis and serves as a pivotal promoter in the progression of DR. Conversely, PPARα activation has demonstrated the ability to down-regulate VEGF, which inhibits the progression of DR [56]. Thus, PPARα emerges as an important player in the management of DR. In addition, thrombomodulin (TM) has been reported to inhibit inflammation in blood vessels [57]. Shiono et al. revealed that THBD, encoding TM, acts as a target gene of PPARα, with PPARα binding close to the transcription start site of THBD in a PPARα-dependent manner, thereby directly upregulating its expression in the DR model [58]. Moreover, in an ischemic model distinct from the diabetic context, PPARα-deficient (Pparα^−/−^) mice exhibited exacerbated choroidal neovascularization (CNV) following laser induction when compared to wild-type CNV mice [59]. Furthermore, PPARα-deficient (Pparα^−/−^) mice exposed to oxygen-induced retinopathy (OIR) demonstrated detrimental consequences, including heightened retinal cell death and intensified glial activation, in contrast to wild-type OIR mice [60]. Notably, the overexpression of PPARα through an adenovirus system mitigated the augmented circulation of endothelial progenitor cells in OIR mice by inhibiting the hypoxia-inducible factor (HIF)-1α pathway. Additionally, mouse brain endothelial cells from PPARα-deficient (Pparα^−/−^) mice displayed significant HIF-1α activation under hypoxic conditions, diverging from wild-type mouse brain endothelial cells [61]. This discovery unveils a novel mechanism involving VEGF, TM, and HIF-1α inhibition for the anti-angiogenic effects of PPARα in DR.

### 3.3. Diabetic Keratopathy

Diabetic keratopathy is an additional ocular complication of diabetes, characterized by corneal neurodegeneration in its early stages [62,63]. One study investigating diabetic keratopathy observed a significant decrease in the protein level of PPARα in the cornea of diabetic rats. This decrease was accompanied by reduced corneal nerve fiber density (CNFD) and nerve sensitivity. However, the use of the PPARα agonist fenofibrate demonstrated the potential to reverse these changes and protect against corneal nerve fiber degeneration and some fatty acids and their metabolites may have similar effects, as they possess polar head groups, a connecting hydrocarbon chain, and a hydrophobic tail, similar to most synthetically made ligands, such as docosahexaenoic acid (DHA) [64]. Additionally, PPARα knockout experiments reveal that Pparα^−/−^ rats exhibited similar outcomes to those induced with diabetes, with a significantly higher incidence of diabetic keratopathy compared to diabetic WT rats [64].

## 4. PPARα Drives Cell Senescence in Alzheimer’s and Chronic Kidney Disease

Senescence is a cellular program that induces a stable growth arrest accompanied by distinct phenotypic alterations, including chromatin remodeling, metabolic reprogramming, increased autophagy, and the implementation of a complex proinflammatory secretome [65]. Aging is characterized by a gradual functional decline. In mammals, aging occurs heterogeneously across multiple organ systems, causing a progressive deterioration that eventually results in tissue dysfunction [66]. Given that lipid disorders like fatty acids and cholesterol metabolic abnormality are critical for cell senescence, the activity of PPARα is closely associated with most aging features [67].

### 4.1. Alzheimer’s Disease

Alzheimer’s disease (AD), accounting for 70% of dementia cases worldwide, is a prevalent form of dementia [68]. The greatest risk factor for AD is aging, and most AD cases are diagnosed in people over 65 years of age [69]. Current evidence suggests that genetic polymorphisms in the PPARα gene, involved in cholesterol and fatty acid (FA) metabolism, are associated with an increased risk of late-onset Alzheimer’s disease (LOAD) [70,71]. Moreover, PPARα knockout mice exhibited impaired long-term memory and hippocampal damage [72,73]. Among the genes implicated in causing AD, the amyloid precursor protein (APP) gene plays a prominent role. It participates in the γ-secretase-mediated processing of APP into amyloid β (Aβ), which is considered the culprit in the disease [68]. One study reveals a three-fold increase in the relative expression of PPARα in the frontal cortex of LOAD, showing a noteworthy negative correlation with APP expression in AD samples but not in normal samples [74]. Moreover, in transgenic mice and cultured cortical cells, human APP expression decreased PPARα expression and its related target genes, while opposite results were observed in APP-silenced cortical networks [74]. Furthermore, PPARα agonists (gemfibrozil and Wy14643) activated PPARα, inducing autophagy in U251 human glioma cells stably expressing human APP and human microglia (HM) cells, which reduced amyloid pathological changes and reversed anxiety symptoms and memory impairment in mice [75].

### 4.2. Chronic Kidney Disease

Aging significantly impacts kidney function and structure, rendering the kidneys highly susceptible to age-related changes [76]. The elderly have a heightened occurrence of chronic kidney disease (CKD), with over one-third of those above 70 experiencing moderate or severe CKD [77]. Renal fibrosis is a prevailing characteristic of all CKD types [78]. Fatty acid oxidation (FAO) predominates as the primary energy source in the kidney’s energy-intensive glomerular region [78]. Dysfunctions in the FAO pathway gain prominence in acute and chronic kidney diseases [79]. PPARα, known for its role in regulating intracellular lipids, has been extensively studied in renal diseases [80]. In an study of aging rats, the expression of PPARα and proteins related to FAO in the renal tubular epithelial region showed a reduction, accompanied by lipids accumulation [81]. Notably, decreased PPARα expression is linked to elevated expression of PPARα-targeted microRNAs [82]. In oleic acid-treated renal epithelial cells, miR-21 effectively suppressed PPARα expression and hindered FAO upon its expression, which worsened lipid accumulation and fibrosis [82]. Moreover, Chung et al. demonstrated that the PPARα agonist (MGY2013) activated PPARα, leading to a reduction in kidney lipid accumulation in aged rats, and effectively reversed the increased collagen I and kidney injury molecule-1 (KIM1) protein levels, which effectively alleviated renal fibrosis and inflammation in aged rats [81].

## 5. PPARα Is a Potential Therapeutic Target for Cancer Treatment

Emerging evidence indicates that cancer is primarily a metabolic disease, involving disturbances in energy production through respiration and fermentation [6]. The genomic instability observed in tumor cells and all other recognized hallmarks of cancer are considered downstream epiphenomena of the initial disturbance of cellular energy metabolism [83]. PPARα could represent a novel strategy for preventing and treating multiple types of cancer, considering that dyslipidemias, obesity, glucose intolerance, and low-grade inflammation are strongly related to an increased risk of cancer [84]. Thus, PPARα could be antitumor molecules associated with cancer cell proliferation, differentiation, and apoptosis [85]. There are also ligand-based studies suggesting that activation of PPAR family members by effective ligands can reduce cellular proliferation and differentiation of cancer cells, providing a theoretical basis for the nutritional regulation of PPARα through strategic approaches [86].

### 5.1. Colorectal Cancer

Colorectal cancer (CRC) ranks as the third most common cancer, according to 2018 statistics from the American Cancer Society [87]. Similarly, the incidence of CRC in China has been steadily increasing in recent years [88]. Emerging evidence indicates that metabolic syndrome is a risk factor for CRC [89,90]. The role of PPARα in colon carcinogenesis has generated conflicting results, likely due to its expression in multiple organs. It has been reported that lower mRNA and protein levels of PPARα are expressed in tumor cells compared to non-tumor cells, and that intestinal PPARα deficiency (Pparα^ΔIE^) enhances azoxymethane (AOM)-induced and dextran sulfate sodium (DSS)-induced colon carcinogenesis [91]. Moreover, in colorectal carcinoma cells characterized by low PPARα mRNA levels, two PPARα agonists (LY171883 and WY14643) mitigated the initial phases of colon tumorigenesis, by suppressing AP-1-mediated transcriptional activation of genes related to the inflammatory response, such as Cox-2 and VEGF [92]. An earlier study also revealed the close relationship between PPARα and inflammatory responses: a lipid metabolite called leukotriene B4 (an inflammatory mediator and natural PPARα ligand) can activate PPARα to establish a negative feedback mechanism, limiting its activity and resolving inflammatory reactions [93]. Concerning colorectal carcinoma cells, an additional PPARα agonist, clofibrate, profoundly inhibits tumor proliferation. It sensitizes colorectal carcinoma cells to chemotherapy drugs in a PPARα-dependent manner, leading to the degradation of the antiapoptotic Bcl2 protein and the induction of autophagy [94]. Furthermore, several reports suggest PPARα ligands as potential chemopreventive agents in colon carcinogenesis. Bezafibrate, a PPARα ligand, is able to suppress AOM and DSS-induced aberrant crypt foci formation in rat colon, decrease intestinal polyp formation in Apc-deficient mice, and inhibit AOM and DSS-induced colon carcinogenesis in mice [46,91]. Given the available evidence, PPARα emerges as a promising candidate for a potential target in treating colorectal cancer.

### 5.2. Kidney Cancer

Renal cell carcinoma (RCC), also known to as kidney cancer, ranks sixth most frequently diagnosed cancer in men and 10th in women worldwide, with a steadily rising incidence rate [95]. Most of the genes typically mutated in renal cell carcinoma have a fundamental role in the regulation of cellular metabolic processes, suggesting dysregulation of the metabolic pathways involved in oxygen, energy, and nutrient sensing as a key feature of RCC carcinogenesis [96]. Notably, PPARα has been confirmed as a potential new RCC target [97]. Additionally, GW6471, a specific antagonist of PPARα, has been reported to decrease fatty acid oxidation (FAO) in the presence of glycolysis inhibition in RCC cells and have a predilection for RCC cells over normal renal tubular epithelial cells [98]. The combined effect of simultaneous administration of PPARα and glycolysis inhibition is likely due to the inhibition of β-oxidation in an FAO-preferential state. In most cancer cells, increased glucose uptake and enhanced glycolytic flux result from hyperactivity of the protein product of the oncogene c-Myc, while PPARα has an opposite effect and its antagonist GW6471 can result a downregulation of c-Myc [99]. Further study found that after 24 h of incubation with GW6471, c-Myc exhibited an inclination towards elevated protein levels in NHK cells, while experiencing a substantial reduction in both RCC cell lines, suggesting that PPARα inhibition mediates its downstream effects via c-Myc and intimating an explanation of the difference in GW6471-mediated lactate levels between RCC and normal epithelial cells. Notably, whether or not concurrent inhibition of glycolysis is achievable, through targeting PPARαt more data have supported that this represents a potential novel and effective therapeutic approach for RCC that fundamentally targets metabolic reprogramming in this tumor [98]. Furthermore, the in vivo activity of GW6471 concerning tumor attenuation is equivalent to that of sunitinib, with no adverse effects and appropriate on-target findings. In addition, PPARα agonists also cause G0/G1 cell cycle arrest as well as induction of apoptosis in kidney cancer cells, which are related to energy metabolism alterations [99]. Thus, PPARα inhibition is a promising new therapy for RCC, acting upon energy metabolism.

### 5.3. Breast Cancer

Breast cancer has been confirmed to have lipid disorders in the tumor microenvironment, which are characterized by altered fatty acid metabolic pathways, including fatty acid transport, *de novo* synthesis, cholesterol synthesis, and activation [100]. PPARα governs the expression of genes related to fatty acid homeostasis, positioning it as a pivotal regulator of lipid metabolism. Due to its impact on lipid metabolism, an escalating number of studies have probed the association between PPARα and breast cancer [101,102]. In breast cancer cells, PPARα contributes to suppressing the activation of fatty acid synthase (FASN), which is associated with poor prognosis and tumor cell metastasis [103,104]. Moreover, PPARα agonists decreased the expression of Acyl-CoA oxidase (ACOX) in breast cancer cells, thereby inhibiting the oxidation of fatty acids and inhibiting the lipogenic pathway [105,106]. Furthermore, PPARα regulates the tumor microenvironment, exerting anti-inflammatory effects and inhibiting angiogenesis by promoting apoptosis [107]. However, whether PPARα can regulate CD36 to prevent the metastasis of breast cancer cells remains to be experimentally confirmed, and further research is needed in this regard. The metabolite 5(S)-hydroxyeicosatetraenoic acid (5-HETE) generated by the 5-lipoxygenase (5-LO) pathway has a growth-promoting effect on breast cancer cells. 5-LO inhibitors upregulate the expression of PPAR-α and γ, and inhibit cell growth when exposed to relevant PPAR agonists. Disruption of the 5-LO signaling pathway mediates growth arrest and apoptosis in breast cancer cells, partially due to induction of PPARs and activation of PPARs with shunted endoperoxides [108]. Another study revealed that DHA induces apoptosis in breast cancer cells by increasing the ratio of cyclic AMP/cyclic GMP levels and promoting Toll-like receptor 4 (TLR4) expression through PPARα [109]. In summary, PPARα’s influence extends to the cell cycle, growth arrest, and apoptosis in both normal and breast cancer cells, and this is achieved by modulating genes within the lipogenic pathway, fatty acid oxidation, fatty acid activation, and the uptake of exogenous fatty acids [46].

### 5.4. Liver Cancer

Hepatocellular carcinoma (HCC) is the most prevalent primary liver cancer among humans, ranking as the third most fatal cancer globally. PPARα agonist fenofibrate induces dose-dependent cell apoptosis in hepatocarcinoma HepG2 cells. Disparities in PPARα levels between human and rodent livers, where human levels are notably lower than in rodents, underlie this distinction. While chronic fenofibrate use leads to rodent liver cancer, elevated fenofibrate concentrations induce cell death in human HepG2 cells by enhancing ROS activity and depleting intracellular glutathione [110]. Moreover, fenofibrate instigates the expression of the *C*-terminal modulator protein within hepatocarcinoma cells. This instigation prompts diminished Akt phosphorylation, which in turn fosters nuclear buildup of the cyclin-dependent kinase inhibitor p27. The resultant augmented p27 accumulation, coupled with the decline of cyclin A and E2F transcription factor 1, culminates in G1 arrest, ultimately facilitating the demise of hepatocarcinoma cells [111].

In the process of hepatocellular carcinoma (HCC) development, long-term presence of liver fibrosis may increase the risk of HCC, and patients with severe liver fibrosis are more likely to progress to HCC. Endocannabinoid-like molecule oleoylethanolamide, acting as a high-affinity ligand for PPARα, improves thioacetamide-induced liver fibrosis in a PPARα-dependent manner [8]. This suggests that PPARα ligands may also possess anti-fibrotic effects.

## 6. Conclusions and Outlook

**Figure 2:**
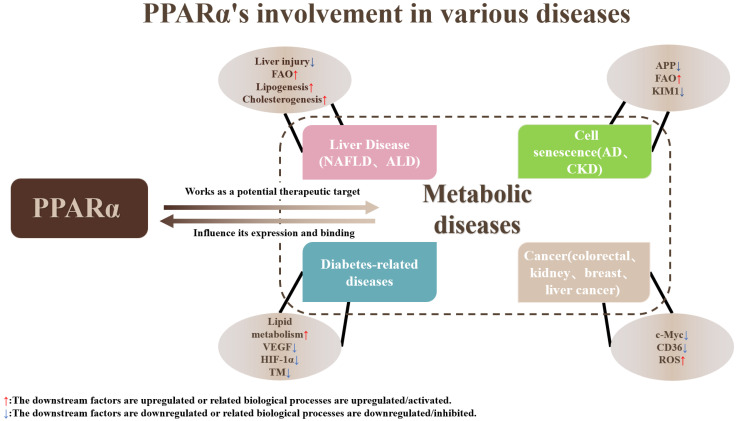
PPARα might serve as a therapeutic target in the pathological processes of liver disease, age-related diseases, diabetes-related diseases, and several cancers by interacting with transcription factors, binding to specific ligands, and participating in cellular physiological processes.

The scope of this review extends beyond providing a concise overview of PPARα’s involvement in various diseases, as shown in Figure 2. As a nuclear receptor, PPARα is widely expressed across multiple organs and exerts a significant influence on diverse disease processes. We are intrigued by the extensive and diverse role of PPARα, which encompasses metabolic disorders and cancer. Despite its broad impact, many aspects of PPARα remain elusive, and numerous details regarding its function remain unknown. Notably, PPARα may exhibit distinct roles in different organs. While liver-specific knockout of PPARα leads to increased plasma cholesterol levels, inflammation, and steatosis, the effects of PPARα knockout in the gut are entirely different. This adds to the enigmatic nature of PPARα’s function.

The prevalence of chronic metabolic diseases and cancer has long been a challenge, and despite advancements in medical science, effective solutions for these ailments are still lacking. Lifespan-related concerns represent a crucial area of investigation within the medical field. In this context, we center our discussion on these aspects and provide an overview of the research progress and accomplishments of PPARα. Notably, studies have highlighted its beneficial effects on liver injury recovery, mitigation of retinal inflammation, and potential role in aging delay [112,113]. These remarkable findings emphasize the necessity for further exploration of PPARα and its potential therapeutic applications.

The strategic exploration and utilization of PPARα holds promise for addressing a broader spectrum of challenging diseases. Currently, metabolic diseases have already benefited from the clinical application of PPARα partial agonists, showcasing their significant therapeutic potential. Notably, fenofibrate, for instance, has emerged as an effective treatment for diabetes [114,115]. Moreover, several PPARα agonists have exhibited promising outcomes in animal studies. In addition to the discussed synthetic ligands, many natural ligands should also be considered, including endogenous metabolites derived from lipid metabolism such as acyl CoAs, oxidized fatty acids, nitrated derivatives of certain fatty acids, and lipoprotein lipolytic products. As natural ligands, they are more readily obtainable and have smaller potential toxic side effects. Therefore, they may be a preferable alternative to expensive synthetically made receptors in treatment strategies. Natural activators of PPARα can also come from exogenous sources, such as those found in dietary nutrients, like dietary ω-3 polyunsaturated fatty acids (docosahexaenoic acid and eicosapentaenoic acid), or from traditional herbal plants [36]. As our understanding of PPARα expands, therapeutic approaches targeting this receptor may offer innovative perspectives for managing related metabolic diseases.

## Data Availability

No new data were created.
